# Aging Biomarkers for Clinical Trials and Drug Discovery

**DOI:** 10.1093/geroni/igab046.014

**Published:** 2021-12-17

**Authors:** Margarita Meer, Raghav Sehgal, Morgan Levine

## Abstract

Developing targeted therapies first requires a working definition of the condition of interest. Unfortunately for aging, this very initial step poses a challenge since chronological age is often not indicative of biological age nor modifiable. This symposium will demonstrate the enormous progress being made towards developing more reliable and valid measures for quantifying biological aging. First, Dr. Albert T. Higgins Chen will show how inaccuracy caused by noise at individual CpG sites can lead to high technical variability in the most widely applied biomarkers of aging—epigenetic clocks. He will further discuss how this can be overcome through novel statistical techniques. Second, Dr. Benoit Lehallier, will discuss plasma proteomic clocks and share insights into their potential roles in Alzheimer's disease and utilization in clinical trials. Third, quantifying the multifactorial aging process can be facilitated by projects incorporating multimodal biomarker data. Pei-Lun Kuo from the Baltimore Longitudinal Study of Aging will present an analysis of longitudinal trajectories of more than 30 phenotypes, which when combined into a single summarized score yield important insights. Fourth, our ability to uncover aging mechanisms and perform drug screens, requires valid and reliable measures that can be applied in vitro. Christopher Minteer who developed in cellulo epigenetic markers will demonstarte how epigenetic aging changes that can be induced in culture shed light on aging in vivo. Finally, a summarizing discussion will be held by Dr. Morgan Levine, an expert in the field.

